# 1404. Tuberculosis and HIV Coinfection: A Review of 135 Cases Experience of the Infectious Diseases Department- CHU Mohamed VI- Marrakech

**DOI:** 10.1093/ofid/ofab466.1596

**Published:** 2021-12-04

**Authors:** Zahid Talibi Alaoui, Mohammed Raiteb, Fatima Ihbibane, Nabila Soraa, noura Tassi

**Affiliations:** 1 Mohammed VI University hospital, infectious disease department, Marrakech, Marrakech-Tensift-Al Haouz, Morocco; 2 Mohammed VI university hospital, microbiology laboratory, Marrakech, Marrakech-Tensift-Al Haouz, Morocco

## Abstract

**Background:**

Tuberculosis is a health problem in Morocco, which is increasingly indicative of human immunodeficiency virus (HIV) infection.

**Objective:**

To determine the epidemiological, clinical and paraclinical, therapeutic and evolutionary aspects of tuberculosis and HIV co-infection.

**Methods:**

we report 135 cases co-infected with HIV and tuberculosis, collected by the infectious diseases department at the Mohammed VI University Hospital in Marrakech. This is a 12-year retrospective study (2007 to 2020) that involved all HIV-infected patients hospitalized for tuberculosis regardless of its location.

**Results:**

The mean age of the patients was 40 years (17-73 years). A male predominance was noted in 69% of cases. In 74.6% of cases, tuberculosis was indicative of HIV infection. Nine patients were receiving antiretroviral (ARV) treatment at the time of the discovery of tuberculosis. There were 24% pulmonary tuberculosis, 25.3% extrapulmonary tuberculosis and 49% disseminated tuberculosis. Tuberculosis was confirmed in 31.7% of cases. At the time of tuberculosis diagnosis, the average CD4 count was 86 cells / mm. Quadruple therapy with isoniazid, rifampicin, pyrazinamide and ethambutol was started in 83% of patients. The average time to start ARVs was 7 weeks. All patients who received ARVs received a combination therapy comprising the combination of 2 nucleoside analogs and one non-nucleoside analog. At the end of our work, the evolution was favorable in 53% of cases, death occurred in 25% of cases, 18.6% of patients were lost to follow-up, two cases of failure and another of relapse. Immune restoration syndrome was noted in 8 cases. Drug toxicity was observed in 24.5% of patients, 73% of which was related to hepato-toxicity of antibacillary drugs.

**Conclusion:**

Tuberculosis is the most common opportunistic infection in people with HIV. Despite the advent of highly active triple therapy, tuberculosis is still a major cause of death in HIV positive people.

**Disclosures:**

**All Authors**: No reported disclosures

